# Supplementary material for: Amyloid β-Peptide Increases Mitochondria-Endoplasmic Reticulum Contact Altering Mitochondrial Function and Autophagosome Formation in Alzheimer’s Disease-Related Models
Source: Cells. 2020 Nov 28;9(12):2552. doi: 10.3390/cells9122552 (PMC7760163; doi:10.3390/cells9122552)
Supplement: Supplementary file 1 [file cells-09-02552-s001.pdf]

Supplementary materials:

Table S1 Clinical data from patients analysed in Figure 1.

Figure S1. Quantifications of MERCS-associated proteins in FAD *post-mortem* brain. Quantification of band intensity from immunoblots in Figure 1 after normalisation to loading control GAPDH: (A) Mfn1 (p = 0.0286), (B) Mfn2 (p = 0.0286), (C) IP3R3 (p = 0.8571), (D) Grp75 (p = 0.6857), (E) VDAC1 (p = 0.8957), (F) VAPB (p = 0.4), (G) PTPIP51 (p = 0.2286), (H) TOM70 (p = 0.0286), (I) TOM20 (p = 0.4857), (J) TIM23 (p = 0.0286), (K) Opa1 (p = 0.6857) and (L) Drp1 (p = 0.4). Comparison between non-dementented (ND, control) and FAD was performed using non-parametric independent Mann-Whitney *U* test. Each dot represents the average of band intensity for each individual patient (n = 4). * $p \leq 0.05$ were considered to be significant.

Figure S2. MERCS and mitochondria ultrastructure are altered in AD mice models and PCN with increased Aβ42. Quantifications of (A) MERCS number (4 months: p = 0.018 App^{NL-F}, p = 0.004 App^{NL-G-F}; 6.5 months: p = 0.045; 10 months: p = 0.002 App^{NL-F}, p = 0.001 App^{NL-G-F}), (B) MERCS length (p = 0.003), (C) mitochondria prolife number (4 months: p = 0.017; 6.5 months: p = 0.037 App^{NL-F}, p = 0.009 App^{NL-G-F}; 10 months: p = 0.042 App^{NL-F}, p = 0.001 App^{NL-G-F}) and (D) mitochondria profile perimeter (6.5 months: p = 0.029; 10 months: p = 0.005 App^{NL-F}, p = 0.025 App^{NL-G-F}) from CA1 electron micrographs Fig. 2A-D. Quantifications of (E) MERCS number (p = 0.009 App^{NL-F}, p = 0.051 App^{NL-G-F}, p = 0.018 $App^{Swe/Lon}$), (F) MERCS length (p = 0.024 App^{NL-F}, p = 0.031 App^{NL-G-F}, p = 0.007 $App^{Swe/Lon}$), (G) mitochondria prolife number (4 months: p = 0.049 App^{NL-F}, p = 0.006 App^{NL-G-F}; 6.5 months: p = 0.004 APP^{NL-F}, p = 0.036 APP^{NL-G-F}; 10 months: p = 0.001 App^{NL-F}, p = 0.044 App^{NL-G-F}) and (H) mitochondria profile perimeter (p = 0.009 App^{NL-F}, p = 0.024 App^{NL-G-F}) from cortex electron micrographs Fig. 2E-G. As before, $APP^{Swe/Lon}$ (red circles), App^{NL-F} (light blue up-triangle) and App^{NL-G-F} (dark blue inverted-triangle) and respective WT control (black rhombus). Solid and dotted lines were used for better visualisation when non-significant but represent the same animals in both (A) and (B). Values represent average of n=3 (WT and $App^{Swe/Lon}$) or n=4 (App^{NL-F} and App^{NL-G-F}) animals and each animal model was compared to the respective age-matched WT. Each animal value was obtained by selecting randomly 3 pictures out of > 100 pictures per animal and all mitochondria and MERCS quantified.

(I) Concentration of extracellular Aβ (pmol/L) of media derived from WT or App^{NL-F} cells with or without γ-secretase inhibitor L685,458 (n = 4-5) (p = 0.0159 Aβ40 WT vs App^{NL-F}; p = 0.0317 Aβ42 WT vs App^{NL-F})

Quantifications of (J) mitochondria profile perimeter and (K) MERCS length from respective electron micrographs from WT or App^{NL-F} 14 DIV derived primary cortical neurons. Each dot represents a measurement of a single cell. $35 \leq n \leq 48$ from 8 (WT) or 5 (App^{NL-F}) independent experiments. Quantification of (L) Mfn2 (p = 0.03357) and (M) VDAC1 band intensity (n = 3-5). p values were obtained by using One-way ANOVA and LSD *post hoc* for (A-H) and non-parametric independent Mann-Whitney *U* test (comparison to WT or – L685,451) in (I-M).

* $p \leq 0.05$, ** $p \leq 0.01$, *** and $p \leq 0.01$ were considered to be significant.

Figure S3. MERCS and mitochondria ultrastructure are altered in WT PCN treated with Aβ42. (A) Representative electron micrographs from WT 14 DIV derived PCN incubated with Aβ42. (B) Representative immunoblot of Mfn2 and 6E10 of WT PCN treated with different concentrations of Aβ42. Quantifications of (C) mitochondria profile number, (D) mitochondria profile perimeter and (E) % of mitochondria surface in contact with ER (p = 0.0302 DMSO vs mAβ42; p = 0.0175 DMSO vs mAβ42+scFvA13). Each dot represents a measurement of a single cell. $33 \leq n \leq 50$ from 8 (WT) or 5 (App^{NL-F}) independent experiments. Quantification of (F) Mfn2 (p = 0.0006) and (G) VDAC1 band intensity (n = 3-7). p values were obtained by using non-parametric independent Mann-Whitney *U* test

(comparison to DMSO). Scale bar corresponds to 500nm, m – mitochondria, arrow – ER, arrow heads – MERCS, n – nucleus. * $p \leq 0.05$ and *** $p \leq 0.01$ were considered to be significant.

Figure S4. Autophagy-associated protein LC3 and p62 as well as MERCS/mitochondria ultrastructure are altered during starvation. Quantifications of autophagy-associated protein from Fig. 3 (A) WT LC3B-I ($p = 0.0009$ Fed vs 1, $p = 0.009$ Fed vs 3), (B) WT LC3B-II ($p = 0.0003$ Fed vs 1.5, $p = 0.0055$ Fed vs 2), (C) WT p62 ($p = 0.0286$), (D) App^{NL-F} LC3B-I, (E) App^{NL-F} LC3B-II ($p = 0.0286$) and (F) App^{NL-F} p62. $3 \leq n \leq 20$ independent experiments and band intensity measure. (G) Representative immunoblots of LC3B of starved 14 DIV PCN derived from WT and App^{NL-F} treated or non-treated with 100nM of autophagosome-lysosome fusion inhibitor Bafilomycin A1 (Baf). Quantifications (H) number of MERCS per mitochondria (WT: p = 0.0273 Fed vs 0.5, $p = 0.0012$ Fed vs 1, p = 0.0096 Fed vs 2, # $p = 0.0002$ 1 vs 1.5; App^{NL-F}: $p = 0.0085$ Fed vs 0.5, p = 0.0060 Fed vs 3), (I) MERCS length (WT: p = 0.0162 Fed vs 0.5, p = 0.00145 Fed vs 2; App^{NL-F}: $p = 0.0325$ Fed vs 1), (J) mitochondria profile perimeter (WT: p = 0.0132 Fed vs 0.5, $p = 0.0009$ Fed vs 1; App^{NL-F}: $p = 0.0354$ Fed vs 0.5, $p = 0.0271$ Fed vs 1) and (K) % mitochondria surface in contact with ER (WT: $p = 0.0032$ Fed vs 1.5, $p = 0.0334$ Fed vs 2.5; App^{NL-F}: $p = 0.0315$ Fed vs 1). Data represents $11 \leq n \leq 48$ from 8 (WT) or 5 (App^{NL-F}) independent experiments. * and # $p \leq 0.05$, ** $p \leq 0.01$ d *** $p \leq 0.01$ were considered to be significant.

Figure S5. Mitochondrial respiration is altered in App^{NL-F} model with increased Aβ42 and WT PCN treated with Aβ42. Comparison of OCR (A) between WT and App^{NL-F} [basal respiration ($p = 0.0159$), ATP production and maximal respiration ($p = 0.0079$)] (n = 4-5) and (B) WT cells treated with Aβ42 (p = 0.0286, n = 3-4). p values were obtained by using non-parametric independent Mann-Whitney U test (comparison to respective Fed condition). * $p \leq 0.05$ and ** $p \leq 0.01$ were considered to be significant.

Table S1 Clinical data from patients analysed in Figure 1.

	ID number	Age of death	Sex	Post-mortem time	Clinical diagonosis	Age of onset	Details
**Ctrl 1**	S3891	82	F	9h	Cardiovascular	-	Moderate arteriosclerosis in brain. No sign of amyloid deposits.
**Ctrl 2**	18491	80	M	16h	Cardiovascular	-	No sign of degeneration or inflammation. Amyloid is not mentioned.
**Ctrl 3**	75090	67	M	21h	Cardiovascular	-	No sign of amyloid deposits.
**Ctrl 4**	6589	68	M	27h	Cardiovascular, pneumonia	-	No sign of amyloid deposits.
**APP Swe 1**	6901	62	M	40h	AD	53	-
**APP Swe 2**	39794	66	M	24h	AD	61	-
**APP Swe 3**	7795	56	M	24h	AD	44	-
**APP Swe 4**	14096	62	F	24h	AD	51	-

Figure S1

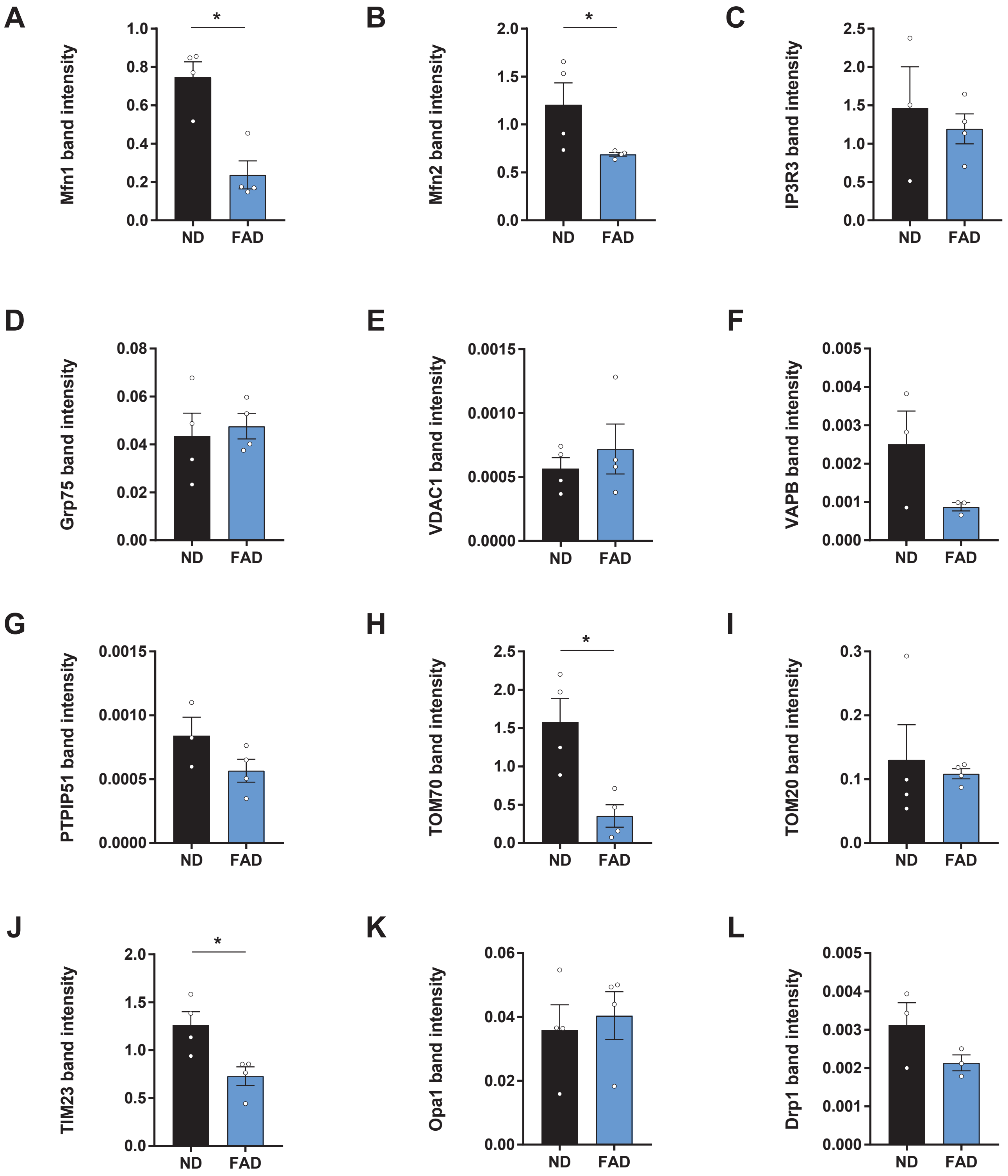
A
Mfn1 band intensity
ND
FAD
*
B
Mfn2 band intensity
ND
FAD
*
C
IP3R3 band intensity
ND
FAD
D
Grp75 band intensity
ND
FAD
E
VDAC1 band intensity
ND
FAD
F
VAPB band intensity
ND
FAD
G
PTPIP51 band intensity
ND
FAD
H
TOM70 band intensity
ND
FAD
*
I
TOM20 band intensity
ND
FAD
J
TIM23 band intensity
ND
FAD
*
K
Opa1 band intensity
ND
FAD
L
Drp1 band intensity
ND
FAD

Figure S2

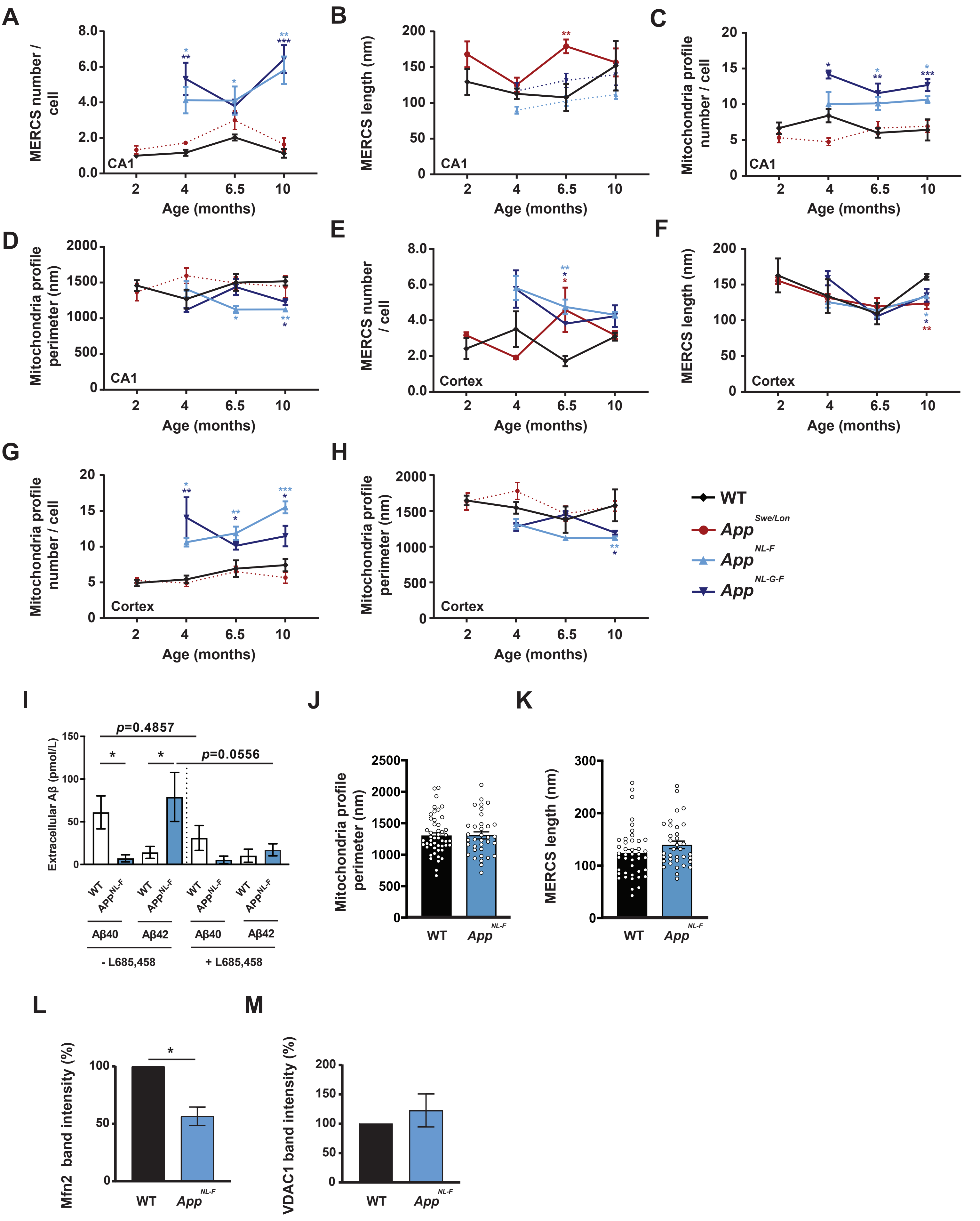

A
MERCS number / cell
CA1
Age (months)
B
MERCS length (nm)
CA1
Age (months)
C
Mitochondria profile number / cell
CA1
Age (months)
D
Mitochondria profile perimeter (nm)
CA1
Age (months)
E
MERCS number / cell
Cortex
Age (months)
F
MERCS length (nm)
Cortex
Age (months)
G
Mitochondria profile number / cell
Cortex
Age (months)
H
Mitochondria profile perimeter (nm)
Cortex
Age (months)
WT
App Swe/Lon
App NL-F
App NL-G-F
I
Extracellular Aβ (pmol/L)
p=0.4857
p=0.0556
WT
APP NL-F
Aβ40
Aβ42
- L685,458
+ L685,458
J
Mitochondria profile perimeter (nm)
WT
App NL-F
K
MERCS length (nm)
WT
App NL-F
L
Mfn2 band intensity (%)
WT
App NL-F
M
VDAC1 band intensity (%)
WT
App NL-F

Figure S3

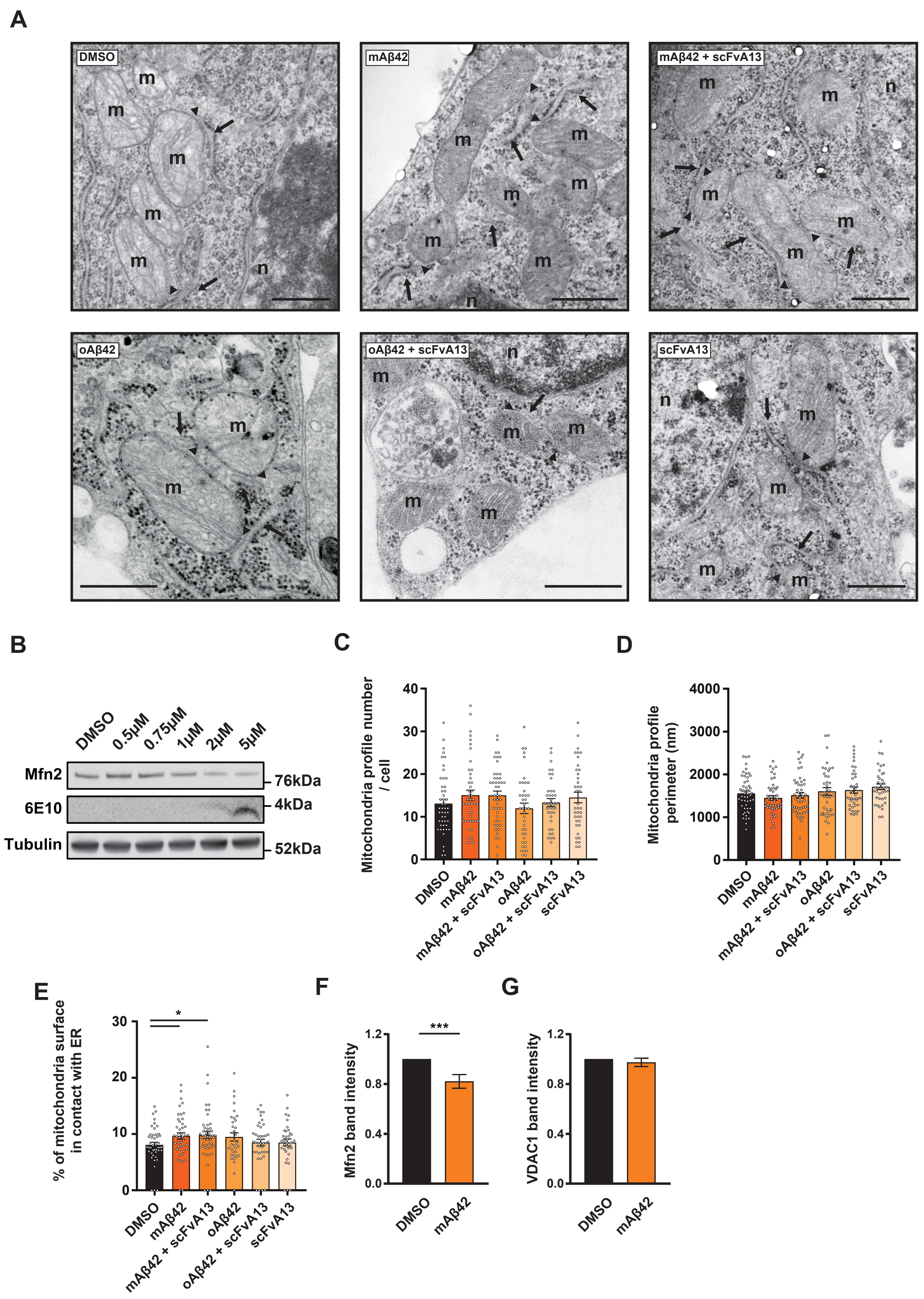
A
DMSO
mAβ42
mAβ42 + scFvA13
oAβ42
oAβ42 + scFvA13
scFvA13
m
n
B
DMSO
0.5μM
0.75μM
1μM
2μM
5μM
Mfn2
6E10
Tubulin
76kDa
4kDa
52kDa
C
Mitochondria profile number / cell
0
10
20
30
40
DMSO
mAβ42
mAβ42 + scFvA13
oAβ42
oAβ42 + scFvA13
scFvA13
D
Mitochondria profile perimeter (nm)
0
1000
2000
3000
4000
E
% of mitochondria surface in contact with ER
0
10
20
30
*
F
Mfn2 band intensity
0.0
0.4
0.8
1.2
***
G
VDAC1 band intensity

Figure S4

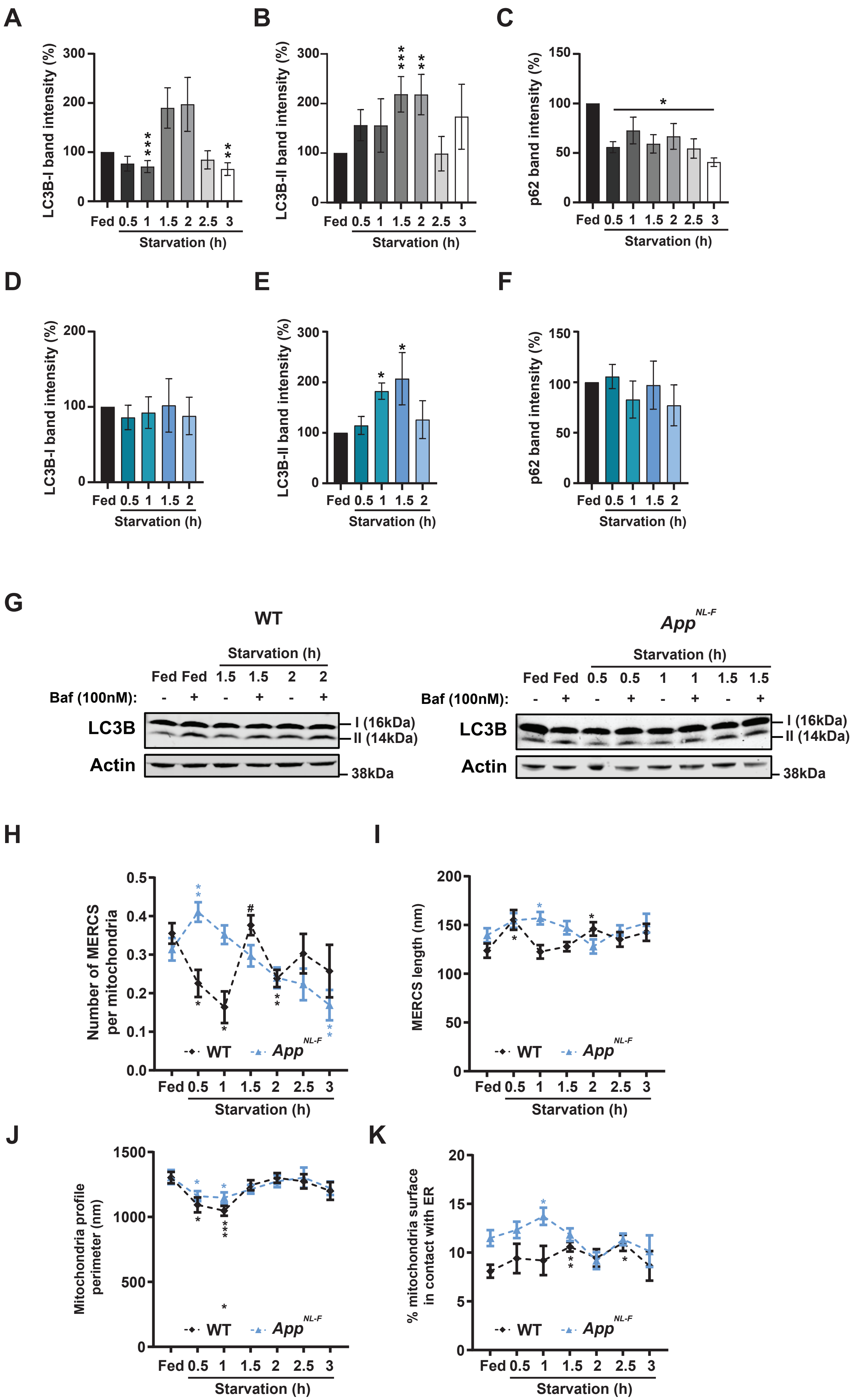
A
B
C
D
E
F
G
H
I
J
K
LC3B-I band intensity (%)
LC3B-II band intensity (%)
p62 band intensity (%)
Starvation (h)
Fed 0.5 1 1.5 2 2.5 3
Fed 0.5 1 1.5 2
WT
App NL-F
Baf (100nM):
Fed Fed 1.5 1.5 2 2
Fed Fed 0.5 0.5 1 1 1.5 1.5
LC3B
Actin
I (16kDa)
II (14kDa)
38kDa
Number of MERCS per mitochondria
MERCS length (nm)
Mitochondria profile perimeter (nm)
% mitochondria surface in contact with ER

Figure S5

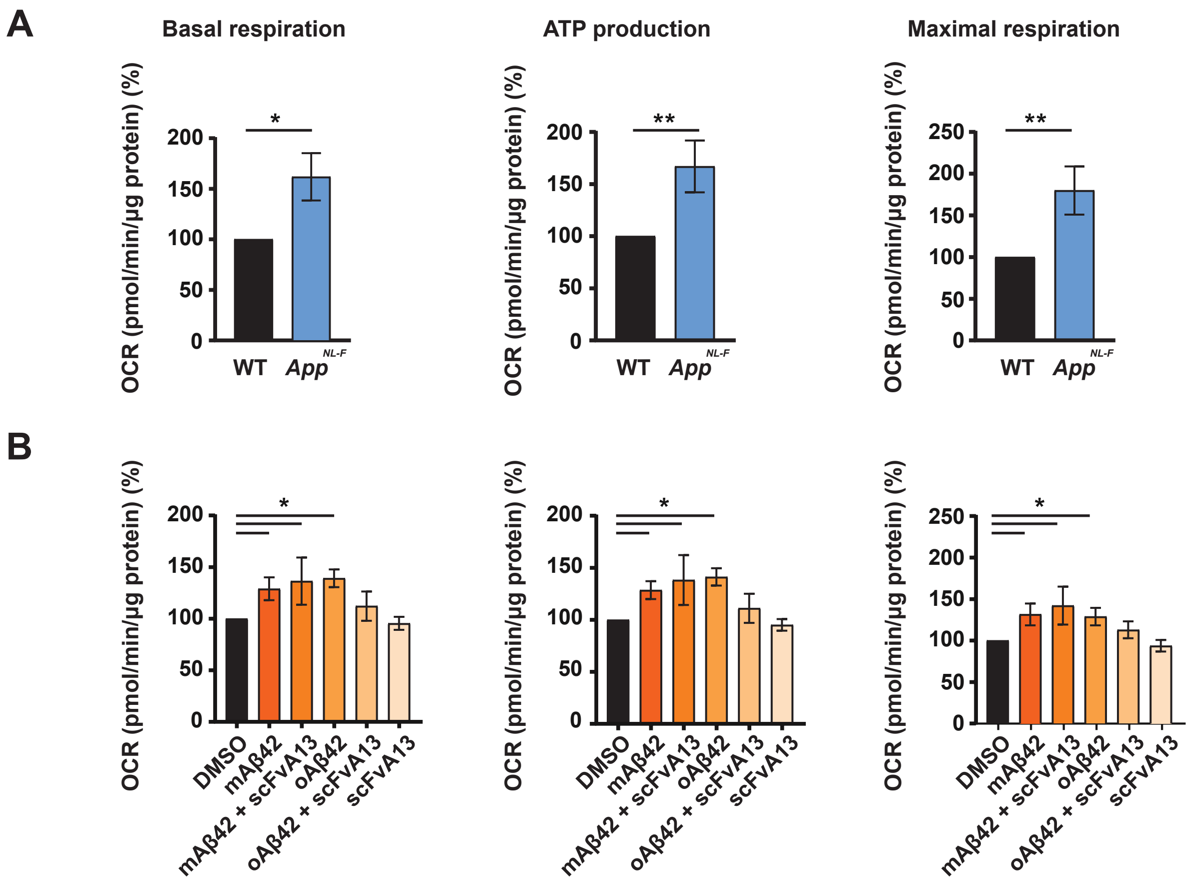

A
Basal respiration
ATP production
Maximal respiration
OCR (pmol/min/µg protein) (%)
WT
App NL-F
*
**
**
B
DMSO
mAβ42
mAβ42 + scFvA13
oAβ42
oAβ42 + scFvA13
scFvA13
